# Community approval required for periconceptional adolescent adherence to weekly iron and/or folic acid supplementation: a qualitative study in rural Burkina Faso

**DOI:** 10.1186/s12978-018-0490-y

**Published:** 2018-03-14

**Authors:** Adélaïde Compaoré, Sabine Gies, Bernard Brabin, Halidou Tinto, Loretta Brabin

**Affiliations:** 1 Clinical Research Unit Nanoro, Institut de Recherche en Sciences de la, Santé, Direction Régionale du Centre-Ouest, Nanoro, Burkina Faso; 2 Department of Biomedical Sciences, Prince Leopold Institute of Tropical Medicine, Antwerp, Belgium; 3 Present address: Medical Mission Institute, Würzburg, Germany; 4 Liverpool School of Tropical Medicine and Institute of Infection and Global Health, University of Liverpool, Liverpool, United Kingdom; Global Child Health Group, Academic Medical Centre, University of Amsterdam, Amsterdam, The Netherlands; 5 Division of Cancer Sciences, Faculty of Biology, Medicine and Health, University of Manchester, Manchester, UK

**Keywords:** Iron supplementation, Adolescents, Adherence, Burkina Faso, Qualitative, Non-pregnant, Pregnant

## Abstract

**Background:**

Iron deficiency remains a prevalent adolescent health problem in low income countries. Iron supplementation is recommended but improvement of iron status requires good adherence.

**Objectives:**

We explored factors affecting adolescent adherence to weekly iron and/or folic acid supplements in a setting of low secondary school attendance.

**Methods:**

Taped in-depth interviews were conducted with participants in a randomised, controlled, periconceptional iron supplementation trial for young nulliparous women living in a rural, malaria endemic region of Burkina Faso. Participants with good, medium or poor adherence were selected. Interviews were transcribed and analysed thematically.

**Results:**

Thirty-nine interviews were conducted. The community initially thought supplements were contraceptives. The potential benefits of giving iron supplementation to unmarried “girls” ahead of pregnancy were not recognised. Trial participation, which required parental consent, remained high but was not openly admitted because iron supplements were thought to be contraceptives. Unmarried non-school attenders, being mobile, were often sent to provide domestic labour in varied locations. This interrupted adherence - as did movement of school girls during vacations and at marriage. Field workers tracked participants and trial provision of free treatment encouraged adherence. Most interviewees did not identify health benefits from taking supplements.

**Conclusions:**

For success, communities must be convinced of the value of an adolescent intervention. During this safety trial, benefits not routinely available in iron supplementation programmes were important to this low income community, ensuring adolescent participation. Nevertheless, adolescents were obliged to fulfil cultural duties and roles that interfered with regular adherence to the iron supplementation regime.

**Trial Registration:**

Trial Registration at clinicaltrials.gov: NCT01210040.

**Electronic supplementary material:**

The online version of this article (10.1186/s12978-018-0490-y) contains supplementary material, which is available to authorized users.

## Background

The nutritional status of adolescents is frequently sub-optimal [1] with a high prevalence of iron deficiency and anaemia [2]. In high prevalence areas, daily iron and folic acid is offered routinely to pregnant women, with intermittent iron supplementation recommended for menstruating women [3]. Achieving high coverage for routine daily iron supplementation in pregnancy has proved difficult and it is uncertain whether, even with intermittent (ie less frequent) dosing, high coverage of non-pregnant women is achievable [4]. Sub-optimal adherence is attributed to side effects which might be reduced by intermittent dosing [5] as well as to operational factors [6]. Reaching unmarried adolescents is challenging as they do not regularly present at health services [7], consequently most adolescent iron intervention studies have been conducted among school attenders [8–11]. Evidence for the effectiveness of iron/folic acid supplementation from community-based studies is weak because of underpowered studies [12]. Without good adherence to the recommended supplementation regime, expected improvements in iron status will not be achieved.

A Cochrane review on iron supplementation (all ages) found inadequate reporting on adherence [13]. Direct observation, the most reliable method, was implemented in a periconceptional, randomised controlled trial (RCT) in Burkina Faso of iron supplementation of never pregnant young women aged 15–24 years, 93% of whom were adolescents (< 20 years) at recruitment [14]. Periconceptional supplementation can provide iron and folic acid in early gestation and prolong the period available for repletion of iron stores before delivery [15]. This was the first periconceptional RCT to recruit nulliparous, mainly adolescent participants, as a high pregnancy rate is more easily achieved by recruiting older, parous women [16]. In our study, field workers (FW) visited homes weekly to deliver weekly iron and folic acid (intervention) or folic acid alone (control). Field records showed varied adherence patterns, with some adolescents periodically absent for “travel,” and others regularly not at home for the arranged weekly visit. The purpose of this qualitative study was to interview participants with varied adherence characteristics in order to better understand factors affecting regular consumption of supplements and encourage adherence.

## Plain English summary

Non-pregnant adolescent girls were followed in an iron and folic acid supplementation trial in rural Burkina Faso, aiming to improve iron stores of young women before their first pregnancy. Families were reliant on subsistence activities, school attendance was low and nutritional status poor. Taking iron supplements regularly is important for improving iron status, but girls were often absent from home at weekly visits when tablets were given. This qualitative study was done to establish why tablets were being missed and to try to encourage adherence. We interviewed 39 young women, with good, medium and poor adherence levels, some of whom had become pregnant during the 18 month supplementation period. Adolescents required approval from male relatives to join the study. Interviews established that many families thought girls were receiving contraceptive pills, which would be generally unacceptable for cultural reasons. Since free treatment was available in the trial, approvals were largely given although most were not allowed to admit they were taking part. Families relied on adolescent domestic labour, and unmarried girls could be sent to work outside the study area, interrupting the iron schedule for indefinite periods. If becoming pregnant before marriage they were also sent away in disgrace. Once married, husbands wanted wives to become pregnant quickly and often stopped the supplements. Adolescents did not relate to the health message that taking iron would benefit a future pregnancy. Maintaining adherence to iron supplements, especially among non-school attenders is operationally challenging and dependent on communities realising benefits from adolescent health interventions.

## Methods

### Background

As giving iron potentially increases malaria and risk of other infections, the RCT assessed the safety and efficacy of iron supplementation of nulliparous women living in a malaria endemic area. In Burkina Faso 52% of young women are married by the age of 18 and 27% have given birth [17]. The population was rural, traditional, predominantly of Mossi tribal origin and reliant on subsistence farming and cattle husbandry. The literacy rate was low in both men and women (~ 23%). Recruitment took place between April 2011 and June 2012 in 30 study villages in the Districts of Nanoro and Yako belonging to the Health and Demographic Surveillance System [18], situated 85 km from Ouagadougou, the capital city. Health care was provided by twelve peripheral health centres and one referral hospital. All healthy, non-pregnant women aged 15–24 years living in these villages were invited to participate. Age and nulliparity were determined at the pre-screening stage. Women with predefined medical conditions were excluded at recruitment by the medical team, as were women with obvious pregnancies, or no menses in the previous three months. Participants (*n* = 1959) were allocated to receive weekly either 60 mg iron and 2.8 mg folic acid (intervention arm) or 2.8 mg folic acid (control arm). Women who remained non-pregnant continued to receive supplements at weekly visits from field workers for18 months, ie up to end assessment. Those who became pregnant continued weekly supplements until their first scheduled antenatal visit (trial primary endpoint at 13–18 weeks gestation), after which routine daily iron and folic acid was provided. In Burkina Faso antenatal care is free. Women attended a second study visit at 33–36 weeks gestation and were followed until delivery. As a safety trial, it was considered unethical to provide no treatment for illness reported at weekly visits and free treatment was made available.

The National Institutes of Health (NIH) funded this trial as part of a competitive grant call on safety of iron supplementation. NIH played no roles in the conduct or analysis of the study. The research was approved by the national Ethical Committee for Health Research and the Institutional Ethical Committee of Centre Muraz in Burkina Faso, and the Ethical Committees of the Liverpool School of Tropical Medicine, United Kingdom, the University of Antwerp, Belgium and the University of Manchester. Trial Registration at clinicaltrials.gov:NCT01210040.

Prior to enrolment the study team visited each village to inform elders and obtain agreement for young women to take part. Thereafter FWs went to each eligible compound to answer questions, emphasising benefits of iron and folic acid for a future pregnancy. At enrolment written consent was given for unmarried participants by parents/guardian or nominated senior women. Individuals also gave their own written consent (or thumb mark) and indicated willingness to be contacted for a future interview. Mean age of participants was 16.8 ± 1.7 years. Median adherence (number of directly observed treatments as a percentage of the number of weeks participating) was 79% (Interquartile range 58%–90%, *n* = 1954), with no difference by trial arm [14].

### Qualitative interviews

Selection was based on adherence level, calculated from weekly FW records. Adherence was classified broadly as “good” (> 50 weekly supplements consumed), “medium” (25–50) or “poor” (< 25), taking into account length of trial participation. The target number of interviews was 15 per category, provided this allowed for data saturation and for inclusion of participants of varying age, marital status and education level. A FW initially contacted participants to arrange an interview with AC, who was known in the villages, having previously conducted focus groups to elicit community views on iron and anaemia [19]. AC explained that the interview was to explore what participants thought of the trial and what had influenced their adherence to supplements. Women generally agreed to be interviewed but if not, an alternative participant with similar characteristics and adherence level was approached. Individual written consent was obtained before each interview.

Most interviews were conducted in Mooré, the local language, in the village or at study headquarters in Nanoro. Individuals were asked to describe their circumstances and expectations of the study. The theoretical perspective was interpretive and derived from Symbolic Interactionism [20] and Identity Theory [21]. The premise was that individual choices (ie taking iron) reflected the several roles individuals fulfilled, albeit choice was constrained by societal and cultural norms and could change over the life course as individuals become tied to their role positions [22]. Interviews were recorded with permission, transcribed into French, which was checked (SG) and then sent to the external collaborator (LB). She advised on subsequent interviews and assessed when interviews yielded no new insights. LB and AC coded the transcripts separately by hand, forming the basis for a field visit, at which emerging themes were agreed. Interviews were analysed without knowledge of trial arm. Additional file S1 provides additional verbatim quotations to support the thematic analysis.

## Results

Interviews were conducted with 39 young women, with one exclusion. Table 1 describes their socio-demographic characteristics and adherence categories. Factors affecting adherence were:

**Table 1:** Socio-demographic characteristics and indicative level of adherence to supplements

N°	Current	School	Occupation	Marital	Delivered	Adhesion
	Age^a^	Grade^b^		Status	Yes/No	Level
1	18	4th Secondary	Student	Single	No	Good
2	18	4th Secondary	Student	Single	No	Medium
3	17	5th Secondary	None	Single	No	Medium
4	17	No Education	Petty Trade	Single	No	Good
5	17	No Education	Petty Trade	Single	No	Good
6	17	No Education	None	Single	No	Medium
7	21	6th Primary	None	Married	No	Medium
8	23	No Education	Petty Trade	Married	No	Medium
9	17	3rd Primary	Sewing	Single	No	Good
10	19	No Education	Sewing	Single	No	Good
11	20	No Education	None	Married	No	Poor
12	20	No Education	None	Married	No	Poor
13	16	No Education	None	Single	No	Poor
14	Excluded ^c^					
15	23	6th Primary	None	Single	No	Good
16	20	2nd Primary	None	Single	No	Good
17	19	6th Primary	None	Single	No	Medium
18	21	3rd Secondary	Student	Single	No	Good
19	19	No Education	None	Married	Yes	Poor
20	21	2nd Secondary	Student	Single	No	Poor
21	17	No Education	None	Single	No	Poor
22	19	No Education	Petty Trade	Married	No	Good
23	16	No Education	None	Single	No	Medium
24	18	4th Secondary	Student	Single	No	Good
25	18	4th Secondary	Student	Single	No	Medium
26	16	No Education	None	Single	No	Good
27	19	No Education	None	Married	Yes	Poor
28	18	No Education	None	Single	No	Medium
29	18	No Education	None	Married	No	Good
30	16	No Education	None	Single	No	Poor
31	22	3rd Secondary	None	Married	Pregnant	Medium
32	18	No Education	None	Married	Yes	Medium
33	18	5th Secondary	Student	Single	No	Good
34	22	No Education	None	Single	No	Poor
35	17	3rd Secondary	None	Single	Yes	Poor
36	24	6th Secondary	Dressmaker	Single	Pregnant	Medium
37	18	No Education	None	Single	No	Poor
38	17	No Education	None	Single	No	Poor
39	16	5th Secondary	Student	Single	No	Good

^a^Reported age at time of interview, between 1 and 2 years after enrolment and commencement of supplementation

^b^Grade attained. French educational system: 6th grade denotes the first grade of Lower Secondary School. Entry into the 2nd (Upper Secondary) grade requires acquisition of Junior Secondary Education Certificate. Primary classes progress in ascending order

^c^Data from this participant was later rejected due to an RCT protocol violation

### Dissatisfaction with taking tablets

Though few side effects were reported, body aches and fever attributed to supplements by ID 38 (poor adherence), which were more suggestive of malaria (for which she was treated) led to her dropping out of the trial. ID 19 (poor adherence) complained of itching, yet when pregnant, took antenatal daily iron with no apparent side effects. ID 17 (medium adherence) refused supplements at menses, saying it caused stomach cramps. Nausea was a problem for ID 15 (good adherence). With a few exceptions (Additional file 1, point a), the intrusiveness of weekly visits was tolerated although participants disliked delays when a FW made several visits in the same compound. Most dissatisfaction was expressed by ID 31 (medium adherence) who complained about the system for reimbursement of health care expenses, her follow-up when she married and moved village, discontinuation of weekly iron supplements when she became pregnant and switched to daily iron as part of routine antenatal care, and her husband’s grievance that the FW did not greet him when she visited.

### Mobility

Periodic absences disrupted adherence. The low level of education (Table 1) meant that respondents mainly attended to domestic chores and/or petty trading. Whereas young men frequently migrated to work in Côte d’Ivoire, girls were generally needed at home. ID 21 (poor adherence) was first sent to Côte d’Ivoire to care for her uncle’s children but had returned to the village. Families living in Côte d’Ivoire tended to send daughters back to stay with the father’s brother. ID 31 (medium adherence) herded cattle and looked after the younger children. Some were sent for domestic work elsewhere within Burkina Faso, and not always in accord with their own wishes. ID 28 (medium adherence) asked to visit her siblings in Côte d’Ivoire or Bobo-Dioulasso but instead was dispatched to the capital, Ouagadougou, to help her paternal uncle. She was recalled after two months to assist her grandmother. ID 34 (poor adherence) was in Bobo-Dioulasso for over a year, caring for her paternal grandfather. After returning from Ivory Coast and joining the study, ID 21 (poor adherence), still aged only 17 years, was sent to Ouagadougou for domestic work, returning home for the harvest season to work in the fields. ID 16 (good adherence), who was absent for two months while staying with her uncle in Ouagadougou, was also recalled, even though she wanted to stay in town where she had fewer chores and more young people for company.

Reliance on older daughters for domestic help probably accounted for the low level of schooling. ID 4 (good adherence) was living in a large household with her paternal uncle because her father was dead and her mother was absent. She has never been sent to school although younger girls in the compound attended. ID 3 (medium adherence) had been obliged to leave school, going to stay with her mother and aunt in Bobo-Dioulasso for five months to sell clothes on the market. She said she disliked housework, did not want to marry but if she did, would choose her own husband. ID 37 (poor adherence) was fearful of the uncle, became anxious when appearing to be idle, and did not attend the health centre when referred by the FW.

School attenders were apt to be mobile in school holidays, when they missed supplements. If they attended high schools in Nanoro and came back to study villages, they could be traced by FWs (eg ID 2 medium adherence), but not if they went elsewhere. Young women who had married into the study area periodically returned to their home villages (eg, ID 8 medium adherence) but generally did not stay for extended periods unless separating from a husband (eg, ID 11, poor adherence). Participants seemed unconcerned about missing doses and often did not inform the FW in advance of an intended move. When asked about her two months absence ID 28 (medium adherence) said,

*No, I wasn’t worried. When I returned I just continued taking it.*

### Individual and community interpretations of the purpose of iron supplementation

The purpose of giving iron supplements as described in briefings and information sheets was not recalled by most interviewees. There were some exceptions. ID 9 (good adherence) was literate, with a brother in University. She, together with her father and brother had read the information sheets and knew the supplements were iron and intended to improve the blood. ID 7 (medium adherence), ID 3 (medium adherence), ID 18 (good adherence), ID 25 (medium adherence) and ID 24 (good adherence) who were literate and/or current students (albeit older and struggling to get good grades) were also better informed. The community could not understand why men were not included or why female participants were nulliparous. It was stated by everyone that the trial was for “*young girls”* and excluded “*women.*”

*Int: Tell me what women’s illnesses you have already had.*

*P: I don’t belong to the category of women.*

*Int: What’s that?*

*P: I’m not a woman. I don’t know.*

*Int: You don’t belong with the women? You belong with the men?*

*P: No, I am a young girl. (ID 34 poor adherence).*

The community initially believed that weekly supplements were contraceptives because iron tablets were strongly associated with pregnancy and antenatal care. This probably explains why, almost without exception, interviewees insisted they never discussed taking part in the trial with anyone – family or friends (Additional file 1, point b). Allowing “*young girls*” to join a trial providing contraception would be controversial so individuals did not readily admit to participation. Thus:

*P: The young men say it is birth spacing medicine.*

*Int: And what do you say?*

*P: We are silent.*

(ID 39 good adherence)

Given this interpretation, why did the community allow “*young girls*” to enrol in the trial?

- Free health care

Whether married or unmarried, young women needed male permission to enrol. Fathers/guardians knew the study provided a bed net and free treatment (Additional file 1, point c) and they sometimes overruled reluctant daughters. A husband’s permission was required when young women married during the trial. Despite free treatment, new husbands wanted their wives to become pregnant and, hearing rumours about contraception, often demurred. ID11 (poor adherence) had married without telling her husband she was enrolled. She was lost to follow-up after marriage but, having left her husband, she requested to rejoin.

- Avoidance of unwanted pregnancy

Unmarried girls who became pregnant represented a community problem and were described as “spoilt.” ID 35 (poor adherence), unmarried, became pregnant, was obliged to leave school and was working in her brother’s shop to earn money. ID 27 (poor adherence) kept her pregnancy secret for as long as possible but eventually fled to the father’s village (contrary to custom), delivered with a traditional attendant and planned to join her “husband” in Côte d’Ivoire.

- Infertility

Three married interviewees with infertility problems were motivated to take supplements associated with pregnancy. ID 29 (good adherence) had been married for three years with no child and her husband had taken a younger wife. He had not objected to her joining the trial, perhaps viewing the contraceptive issue as irrelevant in her case. She became pregnant and attributed her success to the supplement. ID 8 (medium adherence) and ID 7 (medium adherence) did not conceive and were disappointed.

### Opinions on the value of supplementation

Whatever their expectations from the study, and the value accorded to free treatment, very few young women attributed any health benefit to the supplements (iron or folic acid) a themselves (Additional file 1, point d).

## Discussion

Qualitative interviews showed that as “young girls” transitioned to adult women, changing roles and responsibilities often interrupted adherence to iron supplements. Had FWs not been assiduous in tracking participants and negotiating return to the trial, uptake would have been much lower. Adherence was boosted by free medical treatment, whereas the health promotion goal of building iron stores before pregnancy did not enthuse adolescents or communities.

This was the first large community iron supplementation trial of nulliparous young women living in a marginalised rural area. Adherence was closely monitored and qualitative interviews enabled us to interpret observed adherence patterns and explain periodic absences. In rural areas unmarried adolescent girls are an important source of domestic and agricultural labour, but it is difficult to establish whether such changes in residence constitute work or kin fostering [23]. Adolescents can be sent to relatives and strangers, move outside their villages, even between countries. Regionally, families migrate for work and tend to send adolescent girls home [24]. Relocation decisions are usually made by a father or a mother’s brother, often following a critical event such as divorce, when a single parent or new wife, cannot or does not want to cope with a daughter from a previous marriage [25, 26]. Girls reported to us that such moves did not reflect their preferences, and conditions were variable, with the receiving household determining access to health or other benefits. Schooling, unplanned pregnancy and marriage put a brake on mobility. Most girls wanted to go to school yet, when the study was conducted, Burkina Faso had a female literacy rate for 15–24 year olds of 33.1% [27], with just 15.6% of girls in secondary education. Only in 2008 was the minimum age for admission into remunerated employment raised from 14 to16 years in support of obligatory education [28]. Domestic labour and education were catastrophically disrupted by an unplanned pregnancy [29] and, though we could not ascertain their number, many were suspected in cases lost to follow-up. Tradition dictated sending an unmarried pregnant girl away, ideally to a father’s sister. Overtures for reconciliation started after the birth but paternal insistence on an alternative marriage alliance caused family breakdown. Parental control of a daughter’s labour ceased at marriage and the mobility of married Burkinabè adolescents is highly restricted to prevent them running away [30]. Marriage often signalled a negative change in iron adherence.

The rationale for the safety trial was growing evidence that iron supplementation increases malaria infection risk so treatment had to be readily available for safety reasons. Access to health care was valued by adolescents but families may have pushed some girls to participate which could explain some negative attitudes towards the intervention [31]. The research team had not foreseen that supplements would be mistaken for contraceptives. Earlier focus groups indicated only that the function and elemental composition of iron were not well understood and anaemia was not regarded as an adolescent problem [19]. The community struggled with the rationale of giving iron to “young girls” in preparation for pregnancy as iron tablets were a recognised component of antenatal care. The concept of a health intervention to improve adolescent health in its own right has also to be established [32]. As in a school-based study in Dar-es-Salaam, iron supplements were assumed to be contraceptives [11]. In Tanzania this led to study withdrawals, yet enrolment was high in our trial, suggesting that some girls and families were not averse to contraception, even though it could not be discussed or admitted. AC possibly misread frequent silences, as interviewing adolescents, especially when illiterate and economically vulnerable, was challenging. Once aware, the research team did address this misconception and since many adolescents subsequently became pregnant, the notion was dispelled. This may have negatively affected adolescent interest in the supplements.

## Conclusions

The 2030 Agenda for Sustainable Development cannot be met without investment in adolescent health and well-being [33]. Improving nutritional status is an important goal still to be achieved but, if iron supplementation of menstruating girls is perceived to be non-essential, it may be frustrated [34]. Recommended approaches to encourage uptake include strong communication components [11], counselling [35], social marketing with peer educators [36] and integrated adolescent health packages [37]. The evidence base for marginalized and non-school attending populations is lacking [7] as is the evidence for tailored and context-specific nutritional interventions [38]. Integrating prevention of iron deficiency and anaemia with other adolescent interventions may be preferable to stand-alone iron supplementation programmes. Our findings are encouraging in so far as they indicate that delivery of iron supplements as part of a pregnancy prevention strategy may not necessarily meet with parental opposition, if deemed to benefit the wider community. The iron regime (daily or intermittent, length, etc) will need to take account of the specific circumstances likely to interrupt adolescent adherence in each cultural setting, especially among girls who do not progress through secondary education. To ensure improved iron status, the regime must be delivered and adhered to, in line with the recommended guidelines.

## Additional file

Additional File 1: (doc). Additional quotations supporting main themes. (DOCX 15 kb)

## Abbreviations

FW: Field Worker
INT: Interviewer
P: Participant
RCT: Randomised Controlled Trial
