# Supplementary material for: Community approval required for periconceptional adolescent adherence to weekly iron and/or folic acid supplementation: a qualitative study in rural Burkina Faso
Source: Reprod Health. 2018 Mar 14;15:48. doi: 10.1186/s12978-018-0490-y (PMC5852966; doi:10.1186/s12978-018-0490-y)
Supplement: Supplementary file 1 — (doc). Additional quotations supporting main themes. (DOCX 15 kb) [file 12978_2018_490_MOESM1_ESM.docx]

**Supplementary file 1**

Illustrative quotations not cited in the text:

**1a Intrusiveness of weekly visits**

The intrusiveness of a weekly visit risked deterring women from continuing the RCT but only a few women objected openly to the visits. An exception was ID 20, a well-informed higher secondary student, who withdrew from the study because

*Often I get up late. I’m in a hurry to go out, and there she* (the fieldworker) *is. I have to take her products. That doesn’t please me at all. And then often when I get up, I haven’t even eaten, and she is there for me to take the product….I don’t like being disturbed, and I also don’t like having to swallow it.* (ID 20 poor complier)

**1b Refusal to admit study participation**

In the following conversation, the interviewer asks one young woman about a friend whose father had refused permission for recruitment:

*Int: Had you discussed this with her and did she say whether she wanted to participate?*

*P: I never went to discuss it with her.*

*Int: When she sees you, she has never asked you what the medication is that you are taking?*

*P: She has never asked me. (ID 39 good adherence).*

The interviewer asks another participant:

*Int: Do people ask you what medicine you are taking?*

*P: Nobody asks*

*Int: At home? Your parents?*

*P: No. They don’t ask. No. (Id 27 poor adherence)*

*ID 39 (good adherence) was pressed for her reason for taking part:*

*Int: Is it for the free care?*

*P: No*

*Int: Then why?*

*P: (Laughter) No*

*Int: Then why?*

*Silence….*

and

*Int: No-one in the village has come to ask you what you are taking?*

*P: (Laughter) If they ask, we don’t reply.*

*Int: You don’t reply? Why not?*

*P: ……..( laughter) They laugh.*

*Int: They laugh? What do they say?*

*P: They laugh. Are you going to speak and be ridiculed?*

*Int: Why would they laugh? Is it shameful?*

*P: Yes. The men are not going to ask. It’s our friends who are not taking part.*

(ID 4 good adherence)

With regard to contraception, religious beliefs may have been a factor, though no particular denomination was responsible for negative attitudes. Over the 18 month period, approximately a quarter of supplemented women became pregnant, which eventually dispelled these views about contraception.

**1c Importance of free treatment for permission to join the trial**

*P: If you have malaria, they are going to provide care. For vaginal infections, it’s they who provide, and lack of blood as well. All of that, they pay for. Then if you get married, they follow you till you deliver. So I said yes, and my father said that if that was the case, to go.* (ID 31 medium adherence)

Some adolescents were reluctant participants. When asked what happened when she refused to take part, one indicated that it led to problems at home:

*Int: Who told you off?*

*P: My father*

*Int: You were asked to rejoin the study, so why did you return?*

*P: They told me to.*

(ID 30 Poor adherence)

**1d Perceived health benefits of supplements**

The following response was repeated by almost all those interviewed:

*Int: When you take the medication, what changes in your body?*

*P: Nothing has changed (ID 10 good adherence)*

and

*Int: What benefit do you see from taking it?*

*Silence*

*Int: Yes?*

*P: The benefit?*

*Int: Yes*

*P: That’s the problem. (Laughter)* (ID 7 medium adherence)
